# Establishment of well-differentiated camelid airway cultures to study Middle East respiratory syndrome coronavirus

**DOI:** 10.1038/s41598-022-13777-y

**Published:** 2022-06-20

**Authors:** Mitra Gultom, Annika Kratzel, Jasmine Portmann, Hanspeter Stalder, Astrid Chanfon Bätzner, Hans Gantenbein, Corinne Gurtner, Nadine Ebert, Hans Henrik Gad, Rune Hartmann, Horst Posthaus, Patrik Zanolari, Stephanie Pfaender, Volker Thiel, Ronald Dijkman

**Affiliations:** 1 Institute of Virology and Immunology (IVI), Bern and Mittelhäusern, Switzerland; 2 Department of Infectious Diseases and Pathobiology, Vetsuisse Faculty, University of Bern, Bern, Switzerland; 3 Graduate School for Cellular and Biomedical Sciences, University of Bern, Bern, Switzerland; 4 Institute for Infectious Diseases, University of Bern, Bern, Switzerland; 5 Institute of Animal Pathology (COMPATH), Vetsuisse Faculty, University of Bern, Bern, Switzerland; 6 Institute of Animal Pathology, Vetsuisse Faculty, University of Bern, Bern, Switzerland; 7 Department of Molecular Biology and Genetics, Aarhus University, Aarhus, Denmark; 8 Department of Clinical Veterinary Medicine, Ruminant Clinic, University of Bern, Bern, Switzerland; 9 Department for Molecular & Medical Virology, Ruhr University Bochum, Bochum, Germany; 10 Multidisciplinary Center for Infectious Diseases, University of Bern, Bern, Switzerland

**Keywords:** Microbiology, Virology, Viral host response, Viral reservoirs, Virus-host interactions

## Abstract

In 2012, Middle East respiratory syndrome coronavirus (MERS-CoV) emerged in Saudi Arabia and was mostly associated with severe respiratory illness in humans. Dromedary camels are the zoonotic reservoir for MERS-CoV. To investigate the biology of MERS-CoV in camelids, we developed a well-differentiated airway epithelial cell (AEC) culture model for *Llama glama* and *Camelus bactrianus*. Histological characterization revealed progressive epithelial cellular differentiation with well-resemblance to autologous ex vivo tissues. We demonstrate that MERS-CoV displays a divergent cell tropism and replication kinetics profile in both AEC models. Furthermore, we observed that in the camelid AEC models MERS-CoV replication can be inhibited by both type I and III interferons (IFNs). In conclusion, we successfully established camelid AEC cultures that recapitulate the in vivo airway epithelium and reflect MERS-CoV infection in vivo. In combination with human AEC cultures, this system allows detailed characterization of the molecular basis of MERS-CoV cross-species transmission in respiratory epithelium.

## Introduction

Middle East respiratory syndrome coronaviruses (MERS-CoV) emerged in 2012 as the causative agent of severe viral pneumonia in humans. To date, more than 2500 laboratory confirmed cases have been reported, with a case fatality rate of 34%^1^. MERS-CoV is a zoonotic pathogen that is intermittently transmitted from dromedary camels to humans leading to local outbreaks with limited human-to-human transmission. Sero-surveillance indicates that MERS-CoV is enzootic in dromedary camels in the Arabian Peninsula and Africa, and in contrast to humans, only causes a mild respiratory tract infection in camelids^2–4^. Interestingly, the majority of zoonotic infections in humans, as well as higher sero-prevalence of camel exposed workers, are more frequently observed in the Middle East compared to Africa^5,6^. Possible reasons for this pattern might lay in the genetic differences of the virus across the two regions, as well as cultural discrepancies or a lower awareness and surveillance in Africa^5,7–9^. Nevertheless, travel-related cases in 27 countries, as well as nosocomial outbreaks in hospitals, such as in South Korea in 2015 (186 cases and 36 deaths), have been reported and highlight the need for continuous surveillance to mitigate future epidemics^10^.

The anatomical distance of the conducting airways is markedly different between camelids and humans^11^. Concordantly, viral shedding during MERS-CoV infection in humans and camelids is dissimilar, as exemplified by the detection of relatively high levels of MERS-CoV in the upper respiratory tracts of infected camelids, as opposed to humans, in which the infection is restricted to the lower respiratory tract^12–14^. This can be partly explained by the different distribution of the functional receptor—serine exopeptidase Dipeptidyl Peptidase-4 (DPP4)—for MERS-CoV in humans and camelids, which likely influences the limited human-to-human transmission^12,15^. However, the observed discrepancy in the clinical resolution between MERS-CoV infected humans and camelids also suggests that other host determinants, such as the innate immune system, might be of importance.

To facilitate investigations that are focused on the molecular basis underlying the pathogenesis discrepancy of MERS-CoV in humans and camelids, we develop a pseudostratified airway epithelial cell (AEC) culture model for llama (*Llama glama*) and Bactrian camel (*Camelus bactrianus*), analogous to the human AEC culture model. Histological and functional characterization revealed that both camelid AEC culture models closely resemble the in vivo morphology, actively respond to IFNs, and are permissive to MERS-CoV. These three characteristics illustrate that the established camelid AEC model allows detailed future comparative studies on virus-host interactions in human and camelid respiratory epithelium.

## Results

### Establishment of camelid airway epithelial cell cultures

To generate in vitro models that potentially serve as surrogates to characterize MERS-CoV—host interaction at the main replication site in the host reservoir, we sought to establish well-differentiated airway epithelial cell cultures from camelids, analogous to human AEC cultures. Unfortunately, there was no tracheobronchial tissue available from *Camelus Dromedarius* during the entire study period (2014–2021), due to national import and export restrictions. However, as both Bactrian camels and llama are also susceptible to MERS-CoV infection, we chose to isolate primary epithelial cells from post-mortem tissue from tracheobronchial regions of *Camelus bactrianus* (1 donor) and *Lama glama* (2 donors) and propagated them using a pre-established protocol^16^. Following the isolation and expansion, epithelial cells from the old and new world camelids were seeded on semi-permeable cell culture inserts. Once cells reached confluency, the cultures were air-lifted to establish an Air–liquid interface (ALI) to allow for cellular differentiation. During the differentiation process, the development of the camelid AEC cultures was monitored by immunofluorescence analysis with 7-day intervals for a total duration of 28 days.

**Figure 1:**
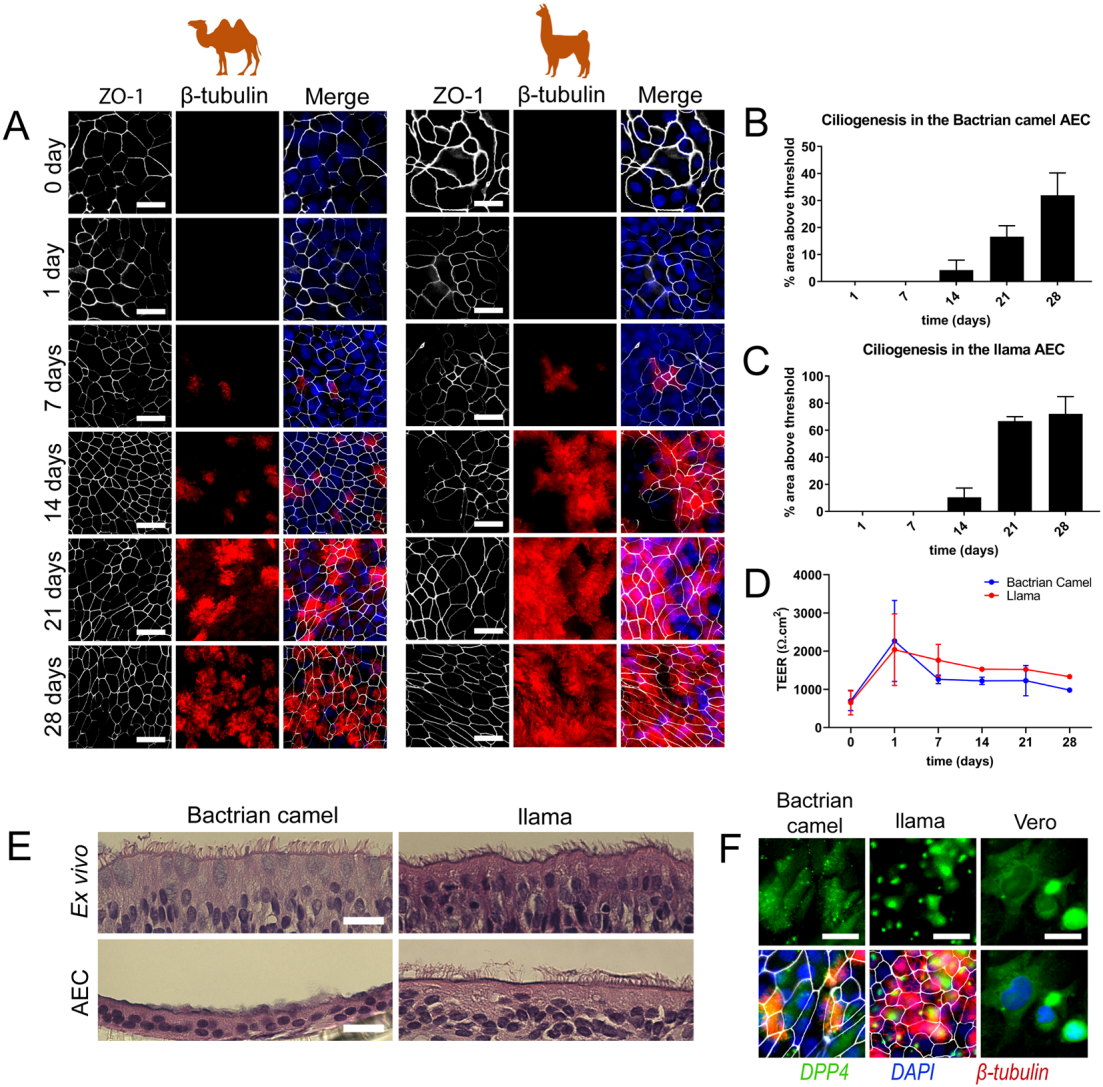
Establishment and characterization of Bactrian camel and llama AEC cultures. (**A**) Immunofluorescence analysis showing the development of tight-junctions (ZO-1, white) and ciliogenesis (β-tubulin, red) in Bactrian camel and llama AEC cultures over time from 1-day to 4 weeks post ALI exposure. The cells were counterstained with DAPI (blue) to visualize the nuclei. (**B,C**) Ciliogenesis quantification of camel and llama AEC cultures overtime, respectively. Ciliation was quantified by measuring the area above a fluorescence intensity threshold of five random images acquired per condition. (**D**) Transepithelial electrical resistance (TEER) measurement of camel and llama AEC cultures overtime during the differentiation. (**E**) Epithelial morphology of ex vivo tissues (upper panel) and well-differentiated camel and llama AEC cultures (lower panel). (**F**) DPP4 expression in well-differentiated camel and llama AEC cultures, with Vero cells as a positive control. Scale bar is 20 µm.

This revealed a progressive ciliary development in both Bactrian camel and llama AEC cultures that reached a plateau after 3 weeks for the llama AEC cultures. For the Bactrian camel AEC cultures, the overall number of ciliated cells was slightly lower (Fig. 1A–C). Tight junction formation in both species seemed to stabilize 2 weeks after ALI establishment as indicated by the trans-epithelial electrical resistance (TEER) measurement and condensed hexagonal architecture of the tight junction marker Zona Occludens 1 (ZO-1) (Fig. 1A,D). In addition to the quantitative measurements, camelid AEC cultures were histologically compared with autologous ex vivo tissue from the corresponding anatomical region. These vertical histologic sections demonstrated that after 28 days of differentiation the camelid AEC cultures formed pseudostratified layer of epithelial cells (Fig. 1E). However, it should be noted that the Bactrian camel AEC cultures exhibited a lesser cell thickness and low number of ciliated cells in comparison to the ex vivo tissue. Nonetheless, combined these results demonstrate that the well-differentiated camelid AEC cultures exhibit morphological properties resembling the Bactrian camel and llama tracheobronchial respiratory epithelium.

### Efficient MERS-CoV replication in Camelid AEC cultures

**Figure 2:**
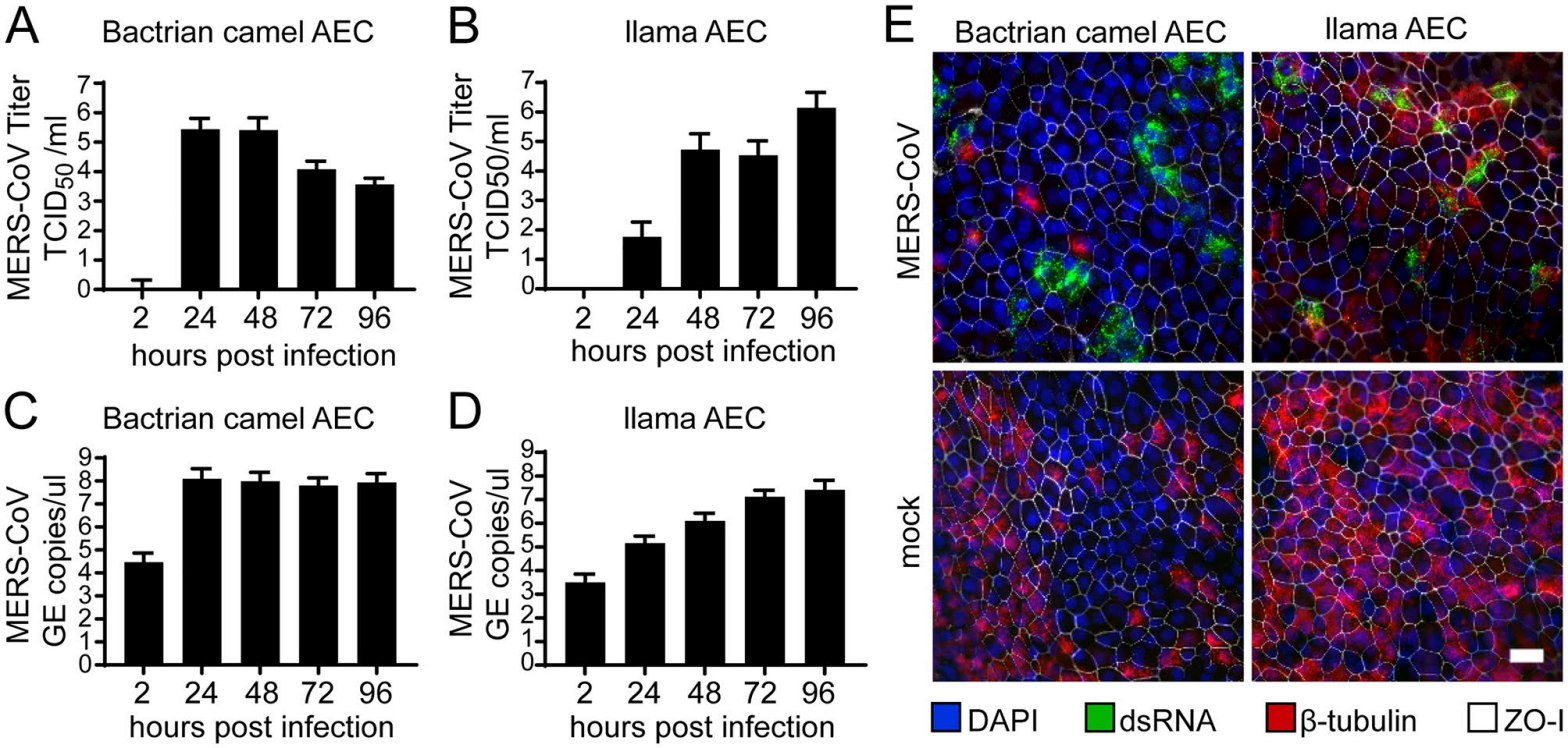
Efficient MERS-CoV replication in camelid AEC cultures. MERS-CoV titer in TCID_50_/ml released from camel (**A**) and llama (**B**) AEC cultures’ apical side from 24 to 96 h post-infection depicted in a log10 scale. Apical virus release in Bactrian camel (**C**) and llama (**D**) AEC cultures measured by quantitative reverse transcription PCR. Data is shown as means and standard deviation from three (**A,C**) or six (**B,D**) independent biological replicates. (**E**) Representative immunofluorescence staining of MERS-CoV-infected camel and llama AEC cultures at 48 post-infection. Double-stranded RNA is shown in green, β-tubulin in red, ZO-I in white and DAPI in blue. Scale bar is 20 µm.

Following the establishment of the camelid AEC cultures, we assessed the expression and distribution of the functional receptor for MERS-CoV in formalin-fixed AEC cultures using a polyclonal antibody against DPP4. As a positive control, we included the Vero E6 cell line, which is known to express DPP4^15,17^. This revealed that DPP4 could readily be detected in both Bactrian camel and llama AEC cultures (Fig. 1F). Of note, the Bactrian camel DPP4 was predominantly distributed at the apical surface of non-ciliated cell populations, while in llama AEC cultures, DPP4 expression is mainly restricted to the apical surface of the ciliated cell population (Fig. 1F). To determine whether both camelid AEC models are susceptible to MERS-CoV, we inoculated well-differentiated Bactrian camel and llama AEC cultures with 4000 PFU of MERS-CoV (MERS-CoV EMC/2012) at 37 °C. At 2 h post-infection (hpi), the apical surface was washed three times with HBSS. Subsequently, virus progeny release was monitored every 24 h for the duration of 96 h by virus titration and qRT-PCR. Interestingly, in both Bactrian camel and llama AEC cultures we observed efficient, albeit dissimilar, MERS-CoV replication profiles. In Bactrian camel AEC cultures MERS-CoV readily reached a plateau at 24 hpi after which the amount of infectious progeny virus declined over time (Fig. 2A,C). In contrast, the overall MERS-CoV replication kinetics in llama AEC cultures was delayed, as MERS-CoV reached the highest titer at 96 hpi (Fig. 2B,D).

Following the replication kinetics, we also assessed the viral cell tropism with immunofluorescence analysis at 48 hpi using an antibody against double stranded RNA (dsRNA), as a surrogate marker for active viral replication. This highlighted that in both Bactrian camel AEC cultures dsRNA was mainly observed in non-ciliated cell populations, similar as in human AEC cultures (Fig. 2E, left panels)^18^. In contrast, in the llama AEC cultures dsRNA-positive cells predominantly overlapped with the cellular ß-tubulin marker used to detect ciliated cells (Fig. 2E, right panels). Altogether, these results demonstrate that both camelid AEC cultures support efficient MERS-CoV replication and indicate that the viral cell tropism coincides with the DPP4 distribution in the AEC models from both camelid species.

### Recombinant IFN inhibits MERS-CoV replication

**Figure 3:**
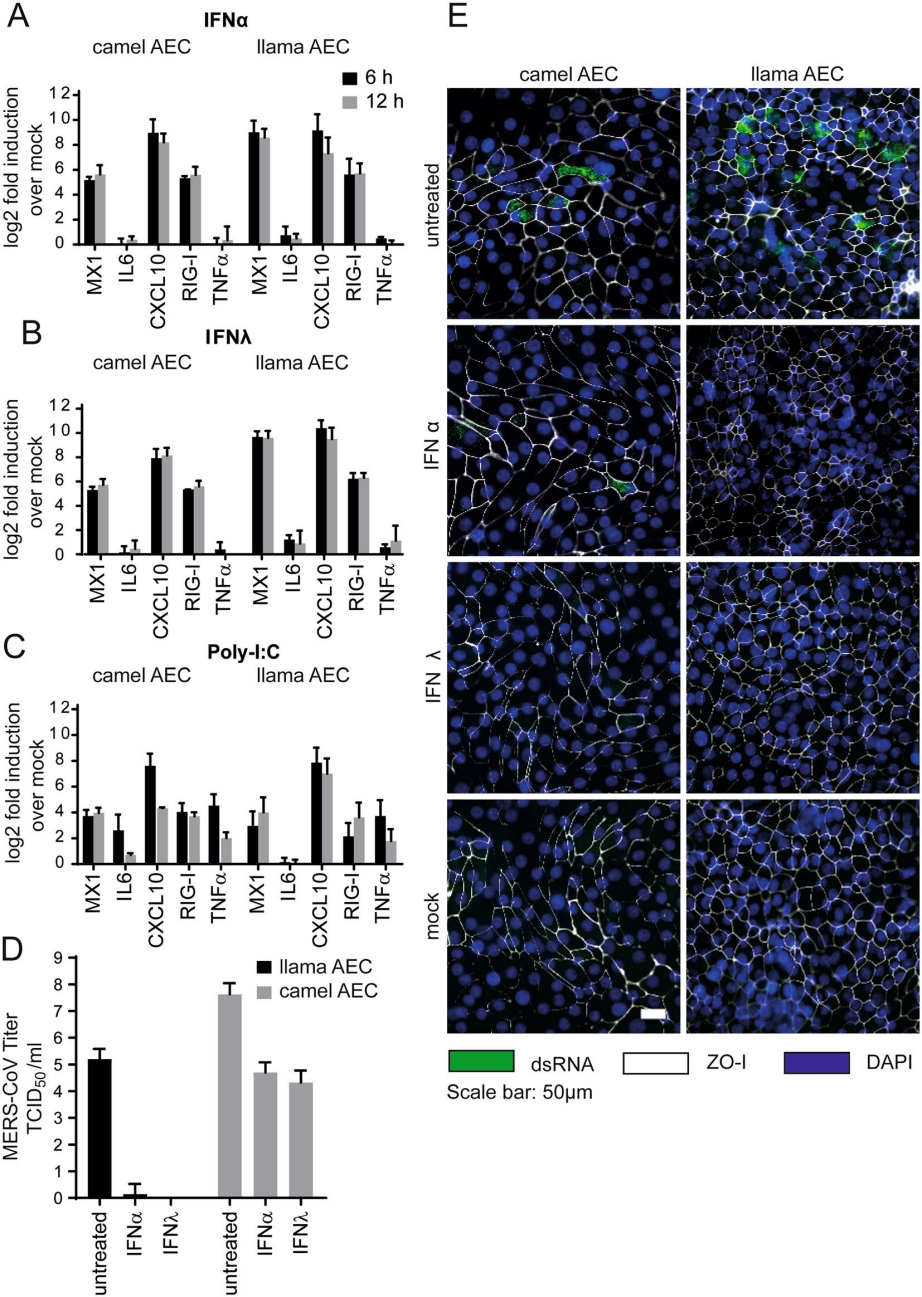
IFN treatment efficiently induces innate immune response and reduces MERS-CoV replication in camelid AEC cultures. MX1, Interleukin-6 (IL6), CXCL10, RIG-I and TNF-α expression in log_2_ fold induction over mock in camel and llama AEC cultures is displayed 6- and 12-h post-stimulation with type I IFN (**A**), type III IFN (**B**), and poly I:C (**C**) treatment. (**D**) MERS-CoV titers in TCID_50_/ml released from camelid AEC cultures’ apical side 48 h post-infection in presence and absence of type I and III IFN pretreatment. Data is shown as means and standard deviation from three independent biological replicates. (**E**) Representative immunofluorescence staining of type I and III IFN pretreated camelid AEC cultures at 48 h post-infection. Double-stranded RNA is shown in green, ZO-I in white and DAPI in blue. Scale bar is 50 µm.

We have previously demonstrated that MERS-CoV replication can be reduced upon exogenous type I and III IFN treatment in human AEC cultures^18^. However, prior to assessing whether MERS-CoV replication can be reduced upon exogenous type I and III IFN treatment in both camelid AEC cultures we first determined whether the cell-intrinsic innate immune system is functional. For this, we stimulated camelid AEC cultures with recombinant pan-species type I IFN, human type III IFN, and synthetic dsRNA (poly-I:C) and analyzed the induction of several host transcripts at 6 h and 12 h post-treatment. This revealed that stimulation by exogenous type I and type III IFNs lead to the induction of canonical ISGs, such as MX1, CXCL10, and RIG-I in both camelid AEC cultures (Fig. 3A,B). Stimulation with poly I:C, a surrogate for active virus replication, resulted in the induction of both ISGs and chemokines (Fig. 3C), signifying that both the sensing and signaling arms of the cell-intrinsic innate immune system seem to be intact in both camelid AEC models.

After having confirmed the functionality of the innate immune system in the camelid AEC cultures, we assessed whether exogenous type I and III IFN treatment could reduce MERS-CoV replication in the camelid AEC cultures, similar as in human AEC cultures. For this, camelid AEC cultures were pre-stimulated with exogenous type I or III IFNs for 18 h prior to MERS-CoV infection. Forty-eight hours post-infection, we observed that in the Bactrian camel AEC cultures apical progeny virus release was reduced by 3.5 to 4 logs by type I and type III IFNs, respectively. Interestingly, for the llama AEC cultures we observed that both type of IFNs markedly reduced MERS-CoV replication (Fig. 3D). These results were corroborated by immunofluorescence analysis at 48 h, in which the number of dsRNA positive cells are reduced in IFN treated cultures compared to untreated cultures (Fig. 3E). This indicates that, like human AEC cultures, MERS-CoV replication can be reduced in both camelid AEC cultures upon type I and III IFN pretreatments. Combined, these results demonstrate that the established camelid AEC models facilitates future experimental work to dissect fundamental virus–host interactions within the camelid respiratory epithelium.

## Discussion

In the current study, we have successfully established well-differentiated AEC cultures from Bactrian camel and llama as surrogate in vitro models to study MERS-CoV in the camelid hosts. We show that the camelid AEC cultures are well-differentiated, possess a functional innate immunity, and support efficient MERS-CoV replication. We observe that the cell tropism and replication kinetic profiles during MERS-CoV infection are dissimilar among the old-world and new-world camelid AEC cultures, resembling the phenotype of MERS-CoV infection in vivo. In addition, we demonstrated that pretreatment with exogenous type I and III IFNs reduces MERS-CoV viral replication in both camelid AEC cultures. Our data suggest that well-differentiated camelid AEC cultures can serve as a more biologically relevant in vitro model that closely resembles the natural site of infection of MERS-CoV in camelids. This approach can help to circumvent the needs of certain animal experiments, especially since experiments using large animals such as camelids are difficult to conduct due to their limited availability and logistical requirements.

During the establishment of the camelid AEC cultures, we observed that the phenotypic appearance of the Bactrian camel AEC cultures did not completely recapitulate the appearance of autologous ex vivo tissue. This observation indicates that further optimization of the cell culture conditions for the Bactrian camel (e.g., seeding density, the concentration of growth and differentiation factors) is likely warranted^16^. Nonetheless, despite this phenotypic disparity, the cell tropism of MERS-CoV in the Bactrian camel AEC cultures coincided with the DPP4 distribution, similar to AEC cultures from Llama or human origin^15^. Furthermore, the replication kinetic profile of MERS-CoV in the Bactrian camel AEC cultures (Fig. 2) was comparable to those observed in vivo by Adney and Colleagues, as such high progeny viral loads within the first 48 hpi were obtained^14^. Finally, both Bactrian camel and llama AEC cultures were responsive to exogenous stimuli, signifying that both the sensing and signalling arms of the cell-intrinsic innate immune system seem to be intact in both camelid AEC models. These key features are often no longer recapitulated in immortalized cell lines due to intrinsic poor biological resemblance^19^. Therefore, despite the current limited phenotypic resemblance of the Bactrian camel AEC cultures compared to autologous ex vivo tissue, our data suggest that well-differentiated camelid AEC cultures can serve as a more biologically relevant in vitro model that closely resembles the natural site of infection of MERS-CoV in camelids.

While dromedary camels are shown as the main zoonotic reservoir for MERS-CoV, susceptibility of both Bactrian camel and llama to MERS-CoV by either natural or experimental infection has been previously reported^14,20–22^. Although both species are genetically closely related, we show that the replication profile as well as cell tropism of MERS-CoV are different in Bactrian camel and llama, in line with those observed results in experimentally infected camels and llamas^13,14,20^. Interestingly, as the natural habitat of Bactrian camels shows a higher overlap to that of dromedary camels, the reservoir for MERS-CoV compared to llamas, MERS-CoV might evolutionarily favor and be better adapted to the Bactrian camel^22^. Differences in MERS-CoV cell tropism can be explained by the distinct MERS-CoV receptor distribution between camel and llama AEC cultures. Nevertheless, differential DPP4 distribution along the respiratory tract of camelids and other ungulates which correlates to their susceptibility has also been described^12,20,23^. However, it was previously shown that the presence of DPP4 alone does not always translate to susceptibility in other animals in vivo, highlighting the importance of investigating the role of other host determinants in the outcome of MERS-CoV infection^20,24^.

In this study, we demonstrate that camelid AEC cultures are responsive to type I and III IFN stimuli and that pretreatment with exogenous IFNs can reduce MERS-CoV replication, similar to previously observed results in primary human AEC cultures^18^. However, despite the close evolutionary relationship between llamas and Bactrian camels, we did observe a species-specific difference in the efficacy of MERS-CoV replication inhibition upon exogenous stimulation with type I and III IFNs. This suggests potential differences in IFN receptor distribution and/or downstream signaling cascades inducing the expression of ISGs tempering MERS-CoV replication. These results, together with the previously reported in vivo data of MERS-CoV infection in alpacas, suggest a dominant role for type I and III IFNs in camelids ^25^. Moreover, since both recombinant type I and III IFNs also efficiently inhibited MERS-CoV in human AEC cultures it would be worth to evaluate the therapeutic potential of IFNs towards MERS-CoV, as well as further investigating MERS-CoV interaction with IFN-related pathways in different host species ^17^. Such analyses can now be performed using the camelid AEC cultures in conjunction with the analogous human AEC cultures, to provide detailed information on crucial virus-host innate immune response dynamics in both natural and zoonotic hosts.

In summary, our results demonstrate that these cultures can serve as a biologically relevant model to characterize fundamental molecular virus-host interactions of MERS-CoV at the natural site of infection in camelids. Altogether, the established camelid AEC culture system, in combination with human AEC cultures, facilitates future detailed characterization of the molecular basis of the pathogenesis discrepancy of MERS-CoV in humans and camelids.

## Materials and methods

### Establishment of camelid AEC cultures

Tracheobronchial epithelial cells from Bactrian camel and llama were isolated from post-mortem tracheobronchial tissue, obtained in collaboration with the veterinary hospital of the University of Bern that euthanized their animals for diagnostic purposes. Isolation and culturing were performed as previously described^16^. Modifications to the composition of the ALI medium were introduced, in which the concentration of the EGF was increased to 5 ng/ml. Both camel and llama ALI cultures were maintained at 37 °C in a humidified incubator with 5% CO_2_. During the development of differentiated camelid ALI cultures (3–4 weeks), media was changed every 2–3 days. During the ALI differentiation stage, inserts were fixed at 7-day intervals from the day of ALI exposure to 4-week post-ALI to monitor the development. TEER resistance was measured every 7 days.

Histological examination of both ex vivo tissues and well-differentiated camelid AEC cultures was done by formalin fixation and staining with haematoxylin and eosin (HE) according to standard histological techniques. The sections were observed and visualized using an EVOS FL Auto 2 imaging system (Thermo Fisher Scientific). Acquired images were processed with Fiji software package v1.53^26^.

### Conventional cell lines

Human hepatoma (Huh7) cell line (kindly provided by Volker Lohmann) was propagated in Dulbecco's Modified Eagle Medium (DMEM), supplemented with 10% heat-inactivated fetal bovine serum, 1% nonessential amino acids, 100 µg/ml of streptomycin, 100 IU/ml of penicillin, and 15 mM of HEPES. Cells were maintained at 37 °C in a humidified incubator with 5% CO_2_. Huh7 cell line was confirmed to be of human origin without contamination, matching the reference DNA of the cell line Huh7 (Microsynth reference, Mic_152021) with 96.7% and the DNA profile of Huh7 (Cellosaurus, RRID:CVCL_0336) with 90%.

### Virus infection

Well-differentiated camelid AEC cultures were infected with 4000 PFU of MERS-CoV (strain EMC/2012, propagated on Huh7 cells diluted in Hanks balanced salt solution (HBSS, Gibco))^18^. The cells were washed with 100 µl of HBSS prior to infection. The virus was inoculated via the apical side. Virus-infected and control AEC cultures were incubated at 37 °C in a humidified incubator with 5% CO_2_. After the inoculation, inoculum was removed, and the apical surfaces were rinsed three times with HBSS, where the third washes were collected as 2 h timepoint. Progeny virus release was monitored with 24-h intervals for a total duration of 96 h, through the application of 100 µl of HBSS onto the apical surface, incubated 10 min prior to the collection time point. The collected apical washes were diluted 1:1 with virus transport medium (VTM) and stored at − 80 °C for later analysis. Following the collection of the apical washes, the basolateral medium was exchanged with fresh ALI medium. Each experiment was repeated with at least three independent biological replicates. For camel, biological replicates were generated from one donor, whereas for llama two different biological donors were used.

### Interferon and poly-I:C stimulation

To analyze the response of camelid AEC cultures to IFN stimulations, both Bactrian camel and llama AEC cultures were treated with recombinant universal type I IFN (100 IU/ml; Sigma Aldrich) or recombinant type III IFN (100 ng/ml) for 6 and 12 h at 37 °C from the basolateral side^27^. For poly-I:C stimulation, AEC cultures were treated with 10 µg poly-I:C (Sigma Aldrich) in 5 0 µl of HBSS from the apical sides for 6 and 12 h at 37 °C. Thereafter, total cellular RNA from the pretreated cells was isolated with the NucleoMag RNA kit (Macherey–Nagel) according to the manufacturer's guidelines on a Kingfisher Flex Purification system (Thermo Fisher Scientific). The quantity of the RNA was determined using NanoDrop (Thermo Fisher Scientific). From the total RNA, cDNA was synthesized using MMLV reverse transcriptase kit (Promega) according to the manufacturers’ protocol with random primers (Promega). Two microliters of diluted cDNA were amplified with SYBR™ Green PCR Master Mix (Thermo Fisher Scientific) according to the manufacturer’s protocol, using primers targeting five different interferon-stimulated genes (ISGs) transcripts (Table 1). GAPDH was used as the reference gene. Measurements and analysis were performed with the Applied Biosystems™ 7500 Fast Dx Real-Time PCR Systems and associated software (Applied Biosystems). Relative gene expression was calculated using the 2^ΔΔCt^ method^28^. Data are shown as fold induction of IFN-treated samples compared to those of untreated controls.

**Table 1:** List of primers used to identify the ISGs in Bactrian camel and llama AEC cultures.

Gene of interest	*Camelus bactrianus*	*Llama glama*	Reference and Genbank accession number
GAPDH	Fw: ATTGTCAGCAACGCCTCCTG		This study, XM_010957730, XM_006210852
	Rev: ACAGTCTTCTGGGTGGCAGT		
Mx1	Fw: AAACAGGGCCCGAGAACAAC		This study, XM_010958347, XM_006204960 (Vicugna pacos)
	Rev: GATGCACGGCCGAATCTTCT		
IL-6	Fw: CTCCATCTGCCCTCCAGGAA		AB107656, AB107647
	Rev: AACTGGACTGAAGGCGCTTG		
CXCL10	Fw: GTCACGGCACCATGAACCAA		This study, XM_010969313, XM_006198241
	Rev: GTGCAGCGTGAAGTTCTGGA		
RIG-1	Fw: AGGGAATGGGTGACCTGGAG	Fw: TGTCCGAGCAGCAGGATTTG	This study, XM_010967358, XM_015249959
	Rev: CACTCAGGACGGAACAAGCC	Rev: CTCGTTGCTGGGATCCATCG	
TNF	Fw: CTACTCCCAGGTCCTCTTCA		Reference^29^, AB178886, AB107646
	Rev: GGTAGTTGGGCATGTTGATC		

To examine the influence of IFN pretreatment on MERS-CoV infection in camelid AEC cultures, the cells were pretreated with type I and III IFNs (100 IU/ml and 100 ng/ml, respectively) 18 h prior to MERS-CoV infection at 37 °C. The basolateral medium containing type I or type III IFN was removed and replaced with medium without exogenous IFN before infection. Untreated Bactrian camel and llama AEC cultures were used as controls. Progeny virus released on the apical sides was collected with 100 µl of HBSS at 48 hpi.

### Virus titration

The 50% tissue culture infectious dose (TCID_50_) per milliliter of supernatant was determined by inoculating Huh7 cells with serially diluted apical washes at indicated hours post-infection. 72 h post-inoculation, cytopathic effect (CPE) was visualized using crystal violet, and TCID_50_ per milliliter was calculated by the Spearman-Kärber algorithm 72 h as previously described^30^.

### qRT-PCR of MERS-CoV

qRT-PCR method was used to determine virus replication. Viral RNA was isolated from the supernatant at indicated hours post infection using the NucleoMag VET Kit (Macherey Nagel) and a Kingfisher Flex Purification System (Thermo Fisher Scientific) according to manufacturer’s guidelines. Extracted RNA was amplified using TaqMan™ Fast Virus 1-Step Master Mix (Thermo Fisher Scientific) according to the manufacturers’ protocol. Primers used for detection of MERS-CoV targeting regions upstream of the E gene (upE) are listed on Table 2 (Genbank accession numbers NC038294 and MG923481)^31^. A serial dilution of in vitro transcribed MERS-CoV RNA (kindly provided by Victor Corman) was used as a reference^31^. Measurements and analysis were performed with the Applied Biosystems™ 7500 Fast Dx Real-Time PCR Systems and associated softwa re (Applied Biosystems).

**Table 2:** Primers used to analyze MERS-CoV replication in Bactrian camel and llama AEC cultures.

Forward upE-Fwd	5′-GCAACGCGCGATTCAGTT-3′
Reverse upE-Rev	5′-GCCTCTACACGGGACCCATA-3′
Probe upE-Prb	5′-FAM-CTCTTCACATAATCGCCCCGAGCTCG-BHQ1-3′

### Immunofluorescence analysis

Cells were fixated with 4% formalin for immunofluorescence analysis. Fixated cells were permeabilized in PBS supplemented with 50 mM NH_4_Cl, 0.1% (w/v) Saponin, and 2% (w/v) Bovine Serum Albumin and stained with a mouse monoclonal antibody against dsRNA (SCIC ONS, clone J2). Alexa-Fluor 488-labeled donkey-anti mouse IgG (H + L) (Jackson Immunoresearch, 715–545–150) was used as a secondary antibody. To visualize the cellular receptor DPP4, a rabbit polyclonal antibody against DPP4 (Abcam, ab28340) was used. Subsequently, Alexa-Fluor 488-labeled donkey-anti rabbit IgG (H + L) (Jackson Immunoresearch, 711–545–152) was used as a secondary antibody. Alexa-Fluor^®^ 647-labelled rabbit anti β-tubulin IV (Cell Signalling Technology, 9F3) and Alexa-Fluor^®^ 594-labelled mouse antibody against ZO-1 (Thermo Fisher Scientific, 1A12) were used to visualize cilia and tight junctions, respectively. Cells were counterstained using 4ʹ,6-diamidino-2-phenylindole (DAPI, Thermo Fisher Scientific) to visualize the nuclei. Images were acquired using an EVOS FL Auto 2 Imaging System, using a 40× air objective. Brightness and contrast were adjusted identically to the correspondin g controls using the Fiji software packages and figures were assembled using FigureJ^26,32^. Quantification of the ciliation was done on five randomized fields of view of β-tubulin-stained inserts acquired with a 40×  objective by measuring cilia-positive area above the threshold.
